# Inflammatory Markers and Outcomes After Liver Resection for Colorectal Liver Metastases

**DOI:** 10.1002/cnr2.70725

**Published:** 2026-11-03

**Authors:** Rider May Cedro, Gilton Marques Fonseca, Fabíola Trocoli Novaes, José Donizeti Meira Júnior, Vagner Birk Jeismann, Fabio Ferrari Makdissi, Jaime Arthur Pirolla Kruger, Fabricio Ferreira Coelho, Paulo Herman

**Affiliations:** ^1^ Liver Surgery Unit, Hospital das Clínicas University of São Paulo School of Medicine São Paulo São Paulo Brazil; ^2^ Bahiana School of Medicine and Public Health Salvador Bahia Brazil

**Keywords:** colorectal neoplasm, hepatectomy, inflammation, liver neoplasms, prognosis

## Abstract

**Background and Objectives:**

The liver is the main site of colorectal metastatic disease. This study evaluated the association of Neutrophil‐to‐Lymphocyte Ratio (NLR), Platelet‐to‐Lymphocyte Ratio (PLR), and Monocyte‐to‐Lymphocyte Ratio (MLR) among patients with colorectal liver metastases (CRLM) undergoing liver resection.

**Methods:**

A cohort of patients undergoing curative‐intent liver resection for CRLM between 2009 and 2020 was evaluated. Association between inflammatory indices and overall survival (OS), disease‐free survival (DFS), and postoperative morbidity and mortality were studied. Subgroup analyses included patients undergoing major hepatectomies (≥ 3 segments) and those receiving preoperative chemotherapy. The indices variation before and after chemotherapy were assessed for OS, DFS, and complete pathological response rates.

**Results:**

After exclusion criteria, 380 patients were selected for the study. No significant associations were found between inflammatory indices and OS or DFS. Lower preoperative PLR was associated with higher postoperative morbidity (*p* = 0.021) after major hepatectomies. Among patients receiving chemotherapy, a greater reduction in PLR was associated with a higher complete pathological response rate (*p* = 0.002).

**Conclusions:**

Although inflammatory indices were not predictive of survival outcomes, PLR was associated with postoperative morbidity and pathological response in specific subgroups; although these findings require validation.

## 1 Introduction

Liver involvement represents the most frequent site of distant metastatic disease in colorectal cancer and is an important determinant of long‐term outcomes. Across the course of the disease, colorectal liver metastases (CRLM) develop in approximately 20%–55% of patients [1, 2].

The biological behavior of CRLM is determined by complex interactions involving tumor characteristics, host immunity, and systemic inflammatory processes [3, 4, 5]. Systemic inflammation has also been implicated in tumor growth, metastatic spread, and the response to anticancer treatment [5, 6].

Blood‐cell–derived inflammatory indices have attracted considerable interest in oncology because they can be obtained routinely and at relatively low cost [7]. NLR, PLR, and MLR are among the most frequently studied indices and capture distinct relationships between circulating inflammatory and immune cell populations. Associations between these measures and clinical outcomes have been reported across several malignancies, although their prognostic relevance has not been consistent across different patient populations or clinical contexts [7, 8, 9, 10, 11, 12].

In patients with CRLM undergoing liver resection, previous studies have reported conflicting associations between inflammatory indices and postoperative and oncological outcomes [8, 13, 14, 15, 16, 17, 18, 19, 20, 21, 22, 23, 24]. Differences in patient selection, timing of metastasis, exposure to preoperative chemotherapy, extent of liver resection, and cutoff definitions may contribute to these inconsistencies. Moreover, most studies have focused primarily on survival outcomes, whereas the potential associations of inflammatory indices with postoperative morbidity and pathological response to preoperative chemotherapy have been less consistently evaluated within the same cohort.

The clinical utility of these biomarkers therefore remains uncertain, particularly regarding whether preoperative inflammatory indices provide meaningful prognostic discrimination and whether changes during systemic treatment are associated with treatment response. Clarifying these associations may help determine whether readily available inflammatory indices have a clinically relevant role in patients undergoing curative‐intent resection for CRLM.

Accordingly, we investigated whether preoperative NLR, PLR, and MLR were associated with overall survival, disease‐free survival, postoperative morbidity, and postoperative mortality in patients undergoing curative‐intent liver resection for CRLM. We further examined these associations in predefined subgroups, including patients undergoing major hepatectomy, and assessed whether changes in these inflammatory indices during preoperative chemotherapy were related to pathological response.

## 2 Material and Methods

### 2.1 Sample

We conducted a retrospective cohort study using data from a prospectively maintained institutional database. The study included consecutive adult patients with CRLM who underwent liver resection with curative intent between 2009 and 2020.

### 2.2 Inclusion and Exclusion Criteria

Patients were eligible if they were adults (≥ 18 years) with CRLM and underwent liver resection with curative intent. Exclusion criteria comprised extrahepatic metastatic disease, previous two‐stage hepatectomy, previous portal vein embolization, and unavailable or insufficient medical records for determining eligibility or study outcomes. Patients who achieved complete pathological response after preoperative chemotherapy remained eligible for analysis. All patients underwent preoperative assessment by a multidisciplinary team.

### 2.3 Pre‐ and Postoperative Variables

The preoperative dataset comprised demographic characteristics, including sex and age, ASA physical status, primary tumor location, timing of CRLM diagnosis, administration of preoperative chemotherapy, pathological T and N stages of the primary tumor, complete blood counts obtained before chemotherapy and before surgery, and prechemotherapy carcinoembryonic antigen (CEA) levels.

Operative and postoperative characteristics included surgical approach (open or laparoscopic), extent of hepatic resection, number and size of liver lesions, resection margin status, pathological response to preoperative chemotherapy, hospital length of stay, and complications occurring within 90 days after surgery. Major hepatectomy was defined as resection of three or more liver segments, whereas resections involving fewer than three segments were classified as minor. Lesions were categorized as unifocal or oligofocal when fewer than four were present and as multifocal when four or more were identified. The largest lesion was categorized according to a 5‐cm threshold. Postoperative complications were graded using the Clavien–Dindo classification. OS and DFS were assessed as the main oncological outcomes.

OS was measured from the date of liver resection until death from any cause or the last documented follow‐up among patients who remained alive. DFS was calculated from liver resection to the first documented disease recurrence, established by imaging or histopathological assessment, or to death from any cause. Patients without either event were censored at their last follow‐up.

Treatment planning was discussed by a multidisciplinary team before surgery. Patients considered for preoperative systemic therapy received oxaliplatin‐based chemotherapy in accordance with the institutional treatment protocol. Tumor response and resectability were reassessed radiologically after 2–4 treatment cycles. Patients with disease considered resectable at restaging subsequently underwent hepatic resection, which was generally performed 2–4 weeks after completion of the last chemotherapy cycle.

Postoperative surveillance consisted of clinical assessments, serum CEA testing, and cross‐sectional imaging with computed tomography or magnetic resonance imaging, performed at 4–6‐month intervals according to the institutional protocol. The median follow‐up was 74.8 months, as estimated by the reverse Kaplan–Meier method. Only one patient (0.3%) was lost during follow‐up.

Primary tumor location was classified as right‐sided colon (cecum to hepatic flexure), left‐sided colon (splenic flexure to the rectosigmoid junction), or rectum. Tumors located in the transverse colon were classified as right‐ or left‐sided according to the surgical approach (extended right or left colectomy, respectively).

According to the E‐AHPBA consensus criteria, CRLM were categorized as synchronous when identified on or before the diagnosis of the primary colorectal tumor, early metachronous when diagnosed within 12 months after the primary tumor, and late metachronous when diagnosed more than 12 months after the primary tumor [25]. All patients who received preoperative chemotherapy were treated with an oxaliplatin‐based regimen according to the institutional protocol.

Histopathological examination of the resected liver specimens was used to evaluate response to preoperative chemotherapy. A complete pathological response was recorded when no viable tumor cells were identified in any of the resected metastatic lesions, with the corresponding tumor sites showing only fibrosis and/or necrosis.

Peripheral blood samples were collected within 1 week before the initiation of systemic chemotherapy and again within 7 days before liver resection as part of routine clinical care. Hematological parameters were analyzed at the institutional central laboratory using an automated analyzer.

Preoperative NLR, PLR, and MLR were derived from the absolute neutrophil, lymphocyte, platelet, and monocyte counts obtained from the blood sample collected before surgery. NLR was calculated as the absolute neutrophil count divided by the absolute lymphocyte count; PLR as the absolute platelet count divided by the absolute lymphocyte count; and MLR as the absolute monocyte count divided by the absolute lymphocyte count. For patients who received preoperative chemotherapy, the change in each index was calculated by subtracting the prechemotherapy value from the preoperative value.

### 2.4 Ethics

This study was approved by the Institutional Ethics Committee (CAPPesq) of the Hospital das Clínicas, University of São Paulo School of Medicine (approval number 5.193.611) and was conducted in accordance with the ethical principles of the Declaration of Helsinki. As this was a retrospective cohort study based on previously collected clinical data, the requirement for informed consent was waived by the Institutional Ethics Committee.

The study was reported in accordance with the Strengthening the Reporting of Observational Studies in Epidemiology (STROBE) recommendations.

The data analyzed in this study were derived from the prospectively maintained institutional database of the Liver Surgery Unit, Hospital das Clínicas, University of São Paulo School of Medicine. This database is part of the institution's ongoing clinical and surgical data collection.

### 2.5 Statistical Analysis

Continuous variables were summarized as means with standard deviations (SDs) or medians with interquartile ranges (IQRs), depending on their distribution, whereas categorical variables were expressed as frequencies and percentages. Comparisons between continuous variables were performed using the Student's *t*‐test or Mann–Whitney *U*‐test, as appropriate. Categorical variables were compared using the chi‐square or Fisher's exact test. All tests were two‐sided, and statistical significance was defined as a *p*‐value < 0.05.

ROC curve analysis was used to determine candidate cutoff values for NLR, PLR, and MLR, with Youden's index applied to identify the corresponding thresholds. Patients were then classified according to these cutoff values. The discriminatory ability of each index was evaluated by the area under the ROC curve (AUC) and its 95% confidence interval (CI). Overall and disease‐free survival were estimated using the Kaplan–Meier method, and survival curves were compared with the log‐rank test.

As an additional exploratory analysis, the authors evaluated the relationship between preoperative inflammatory indices (NLR, PLR, and MLR) and patient age, sex, and the timing of colorectal liver metastases. Age was assessed both as a continuous variable and using the cohort median of 62 years as a cutoff. Comparisons between inflammatory indices and sex were performed using the Mann–Whitney *U* test, while differences according to E‐AHPBA metastatic timing (synchronous, early metachronous, and late metachronous) were assessed using the Kruskal–Wallis test. Spearman rank correlation was used to assess the association between age and inflammatory indices. Additional exploratory Cox proportional‐hazards models and interaction analyses were performed to evaluate whether age, sex, or metastatic timing modified the association between inflammatory indices and overall or disease‐free survival. Detailed results of these additional analyses are provided in the Supporting Information.

## 3 Results

### 3.1 Baseline Characteristics

A total of 492 consecutive patients with CRLM were identified between 2009 and 2020. Patients who underwent R2 resection were not considered eligible. Based on the predefined exclusion criteria, 25 patients undergoing two‐stage hepatectomy, 70 with concomitant extrahepatic metastatic disease, 10 who underwent portal vein embolization, and 7 with unavailable medical records were excluded. The final study cohort therefore comprised 380 patients. Baseline characteristics are presented in Table 1.

**TABLE 1:** Baseline characteristics.

	*n* = 380	*n* (%)	
Sex	Female	171 (45.0)	
	Male	209 (55.0)	
Age at surgery (years)	Mean (SD)		60,9 (10.2)
	Median (P_25_‐P_75_)		62 (55–68)
ASA	I	65 (17.1)	
	II	282 (74.2)	
	III	33 (8.7)	
Location of the primary neoplasm	Right colon	60 (15.8)	
	Left colon	203 (53.4)	
	Rectum	117 (30.8)	
Timing of liver metastases	Synchronous	56 (14.7)	
	Early metachronous	214 (56.3)	
	Late metachronous	110 (28.9)	
Preoperative chemotherapy^1^	No	103 (27.2)	
	Yes	276 (72.8)	
T of the primary tumor^2^	T1‐T2	29 (8.0)	
	T3‐T4	333 (92.0)	
N of the primary tumor^3^	N0	166 (45.2)	
	N+	201 (54.8)	
Surgical approach	Open	271 (71.3)	
	Laparoscopic	109 (28.7)	
Type of liver resection	Minor Hepatectomy	284 (74.7)	
	Major Hepatectomy	96 (25.3)	
Number of resected nodules	1–3 nodules	292 (76.8)	
	4 or more nodules	88 (23.2)	
Size of the largest resected nodule (cm)^4^	≤ 5 cm	297 (83.9)	
	> 5 cm	57 (16.1)	
Pathological pattern	Colorectal cancer metastasis	355 (93.4)	
	Complete pathological response	25 (6.6)	
Surgical margins^5^	Free (R0)	321 (90.9)	
	Microscopically positive margin (R1)	32 (9.1)	
CEA (ng/dL)^6^	< 20	255 (67.3)	
	≥ 20	124 (32.7)	

Abbreviations: ASA, American Society of Anesthesiologists; CEA, carcinoembryonic antigen; P_25_, 25th percentile; P_75_, 75th percentile; SD, standard deviation.

^1^ 1 case without information on preoperative chemotherapy.

^2^ 18 cases without information on T stage.

^3^ 13 cases without information on N stage.

^4^ 26 cases without information on Size of the largest resected nodule.

^5^ 27 cases without information on surgical margins.

^6^ 1 case without information on CEA.

In the overall cohort, the median preoperative counts were 3200/μL (IQR, 2340–4200/μL) for neutrophils, 1600/μL (IQR, 1200–2125/μL) for lymphocytes, and 184 000/μL (IQR, 149000–229 000/μL) for platelets. Detailed cell counts for the overall cohort and the principal clinical subgroups are provided in Table 2.

**TABLE 2:** Baseline absolute neutrophil, lymphocyte, and platelet counts according to the main analyzed subgroups.

Subgroup	*n*	Neutrophils	Lymphocytes	Platelets
Overall	380	3.200 (2.340–4.200)	1.600 (1.200–2.125)	184.000 (149.000–229.000)
*Sex*				
Female	171	3.200 (2.220–3.930)	1.700 (1.300–2.300)	195.500 (160.250–246.000)
Male	209	3.245 (2.400–4.475)	1.520 (1.100–2.100)	173.500 (140.250–212.750)
*Preoperative chemotherapy* ^*1*^				
Yes	276	3.150 (2.215–3.895)	1.600 (1.185–2.145)	173.500 (141.750–217.000)
No	103	3.700 (2.785–4.900)	1.690 (1.200–2.100)	210.000 (163.000–251.000)
*Type of hepatectomy*				
Major	96	3.200 (2.388–4.050)	1.600 (1.152–2.178)	178.500 (146.250–218.250)
Minor	284	3.220 (2.330–4.215)	1.600 (1.200–2.100)	184.500 (150.750–231.250)
*Timing of liver metastases*				
Synchronous early	56	3.550 (2.582–4.700)	1.650 (1.192–2.170)	182.500 (152.500–237.500)
Metachronous late	214	3.050 (2.200–3.730)	1.600 (1.190–2.100)	178.500 (148.000–222.500)
Metachronous	110	3.485 (2.645–4.675)	1.605 (1.200–2.152)	188.500 (155.250–229.250)

*Note:* Absolute neutrophil, lymphocyte, and platelet counts were obtained from the preoperative complete blood count (cells/μl).

Abbreviation: IQR, interquartile range.

^1^ 1 case without information on preoperative chemotherapy.

### 3.2 Optimal Cut‐Offs for NLR, PLR, and MLR

ROC analyses were performed to identify candidate thresholds for NLR, PLR, and MLR in relation to mortality and recurrence after liver resection. The thresholds selected according to Youden's index were subsequently used for the categorical analyses. For mortality, the cutoff values were 1.40 for NLR, 106.33 for PLR, and 0.28 for MLR, with corresponding AUCs of 0.499, 0.521, and 0.517, respectively. For recurrence, the cutoff values were 1.56 for NLR, 102.26 for PLR, and 0.22 for MLR, with corresponding AUCs of 0.531, 0.489, and 0.502, respectively. The complete ROC analysis results are presented in Table 3.

**TABLE 3:** ROC curve analysis and optimal cutoff values for NLR, PLR, and MLR according to mortality and recurrence.

	Cut‐off	Sensitivity	Specificity	Area under the curve	*p*
Mortality					
NLR	1.40	79.3%	20.0%	0.499	0.974
PLR	106.33	72.3%	29.7%	0.521	0.488
MLR	0.28	62.6%	39.5%	0.517	0.577
Recurrence					
NLR	1.56	77.6%	27.1%	0.531	0.322
PLR	102.26	75.7%	28.8%	0.489	0.729
MLR	0.22	80.3%	22.4%	0.502	0.944

Abbreviations: MLR, Monocyte‐to‐Lymphocyte Ratio; NLR, Neutrophil‐to‐Lymphocyte Ratio; PLR, Platelet‐to‐Lymphocyte Ratio.

### 3.3 Association of Inflammatory Indices With OS and DFS

The median OS was 69.5 months. The estimated OS rates at 1, 3, and 5 years were 88.6%, 67.6%, and 53.0%, respectively. The estimated DFS rates at 1, 3, and 5 years were 59.4%, 39.2%, and 36.7%, respectively. No statistically significant differences in OS or DFS were observed according to preoperative NLR, PLR, or MLR categories (Figure 1).

**FIGURE 1:**
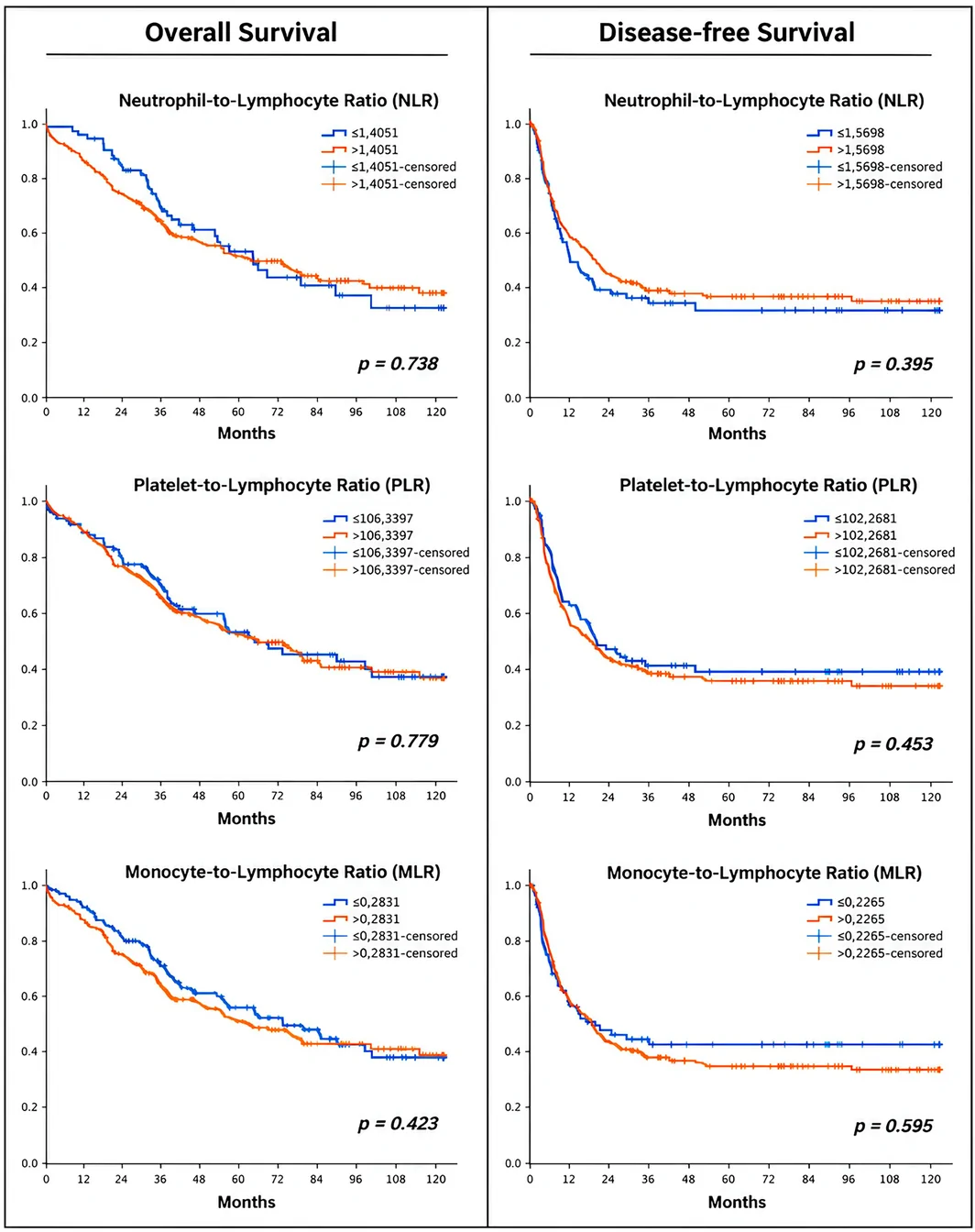
Overall survival and disease‐free survival for patients with colorectal liver metastases according to the Neutrophil‐to‐Lymphocyte Ratio (NLR), Platelet‐to‐Lymphocyte Ratio (PLR), and Monocyte‐to‐Lymphocyte Ratio (MLR).

### 3.4 Subgroup Analysis

Among patients undergoing major hepatectomy, a significant association was observed between a preoperative PLR ≤ 106.33 and higher rates of severe postoperative morbidity (Clavien–Dindo Grades III–IV), compared with patients with a PLR > 106.33 (*p* = 0.021). No significant association was observed between NLR, PLR, or MLR and postoperative mortality in this subgroup (Table 4). Patients with Clavien–Dindo Grade V were excluded from the analysis of morbidity severity.

**TABLE 4:** Postoperative morbidity and mortality for subgroup of patients undergoing major hepatectomies.

		Morbidity		*p*	Mortality		*p*
		CD 0‐II	CD III‐IV		CD 0‐IV	CD V	
		*n* = 79; *n* (%)	*n* = 7; *n* (%)		*n* = 86; *n* (%)	*n* = 10; *n* (%)	
NLR	≤ 1.40	9 (12.3)	1 (20.0)	0.506	10 (12.8)	1 (10.0)	0.999
	> 1.40	64 (87.7)	4 (80.0)		68 (87.2)	9 (90.0)	
PLR	≤ 106.33	18 (24.7)	4 (80.0)	**0.021**	22 (28.2)	4 (40.0)	0.473
	> 106.33	55 (75.3)	1 (20.0)		56 (71.8)	6 (60.0)	
MLR	≤ 0.28	24 (32.9)	3 (60.0)	0.334	27 (34.6)	3 (30.0)	0.999
	> 0.28	49 (67.1)	2 (40.0)		51 (65.4)	7 (70.0)	

*Note:* Percentages were calculated based on available data for each variable. For NLR, PLR, and MLR, data were available for 73 patients in the CD 0–II group, 5 patients in the CD III–IV group, and 78 patients in the CD 0–IV group. Missing data were excluded from the respective analyses. Bold values indicate statistically significant results (*p* < 0.05).

Abbreviations: CD, Clavien–Dindo Classification; MLR, Monocyte‐to‐Lymphocyte Ratio; NLR, Neutrophil‐to‐Lymphocyte Ratio; PLR, Platelet‐to‐Lymphocyte Ratio.

Among the 276 patients who received preoperative chemotherapy, changes in NLR, PLR, and MLR between the prechemotherapy and preoperative periods were evaluated in relation to pathological response, OS, and DFS. No significant associations were observed between changes in NLR, PLR, or MLR and OS or DFS. Similarly, prechemotherapy values of NLR, PLR, and MLR were not significantly associated with complete pathological response.

In contrast, the change in PLR was significantly associated with complete pathological response (*p* = 0.002). Among patients with ΔPLR ≤ −65.74, 20.8% achieved a complete pathological response, compared with 6.8% of patients with ΔPLR > −65.74 (Table 5).

**TABLE 5:** Pathological response according to the Neutrophil‐to‐Lymphocyte Ratio (NLR), Platelet‐to‐Lymphocyte Ratio (PLR) and Monocyte‐to‐Lymphocyte Ratio (MLR) variation in patients with CRLM who underwent preoperative chemotherapy.

	Complete pathological response	Colorectal cancer liver metastasis	*p*
	*n* = 25	*n* = 251	
	*n* (%)	*n* (%)	
ΔNLR			0.126
≤ −1.13	8 (15.1)	45 (84.9)	
> −1.13	17 (8.2)	191 (91.8)	
ΔPLR			**0.002**
≤ −65.74	11 (20.8)	42 (79.2)	
> −65.74	14 (6.8)	193 (93.2)	
ΔMLR			0.134
≤ −0.09	7 (15.6)	38 (84.4)	
> −0.09	18 (8.3)	198 (91.7)	

*Note:* Missing data were excluded from the respective analyses. ΔNLR, ΔPLR, and ΔMLR were available for 261, 260, and 261 patients, respectively. Bold values indicate statistically significant results (*p* < 0.05).

Abbreviations: CRLM, colorectal liver metastasis; MLR, Monocyte‐to‐Lymphocyte Ratio; NLR, Neutrophil‐to‐Lymphocyte Ratio; PLR, Platelet‐to‐Lymphocyte Ratio.

In the same subgroup of patients who received preoperative chemotherapy, four patterns of inflammatory index evolution were defined according to prechemotherapy and preoperative values: high‐to‐low, low‐to‐low, low‐to‐high, and high‐to‐high. No significant differences in OS or DFS were observed among these four evolution patterns. Furthermore, the evolution patterns were not significantly associated with complete pathological response.

### 3.5 Additional Analyses

Additional exploratory analyses were performed to evaluate the relationship between preoperative inflammatory indices and patient age, sex, and the timing of colorectal liver metastases.

Age was not significantly correlated with NLR or MLR, whereas PLR showed a weak inverse correlation with age. When patients were stratified according to the median age of 62 years, NLR and MLR did not differ significantly between age groups, while PLR was lower among patients aged ≥ 62 years (*p* = 0.050).

NLR and MLR were significantly higher in male than in female patients, whereas PLR did not differ significantly according to sex. In analyses according to E‐AHPBA metastatic timing, no significant differences in NLR, MLR, or PLR were observed among patients with synchronous, early metachronous, or late metachronous metastases.

Additional exploratory Cox models and interaction analyses evaluating age, sex, and metastatic timing as potential effect modifiers are presented in the Supporting Information.

## 4 Discussion

In this cohort of 380 patients undergoing curative‐intent liver resection for CRLM, preoperative NLR, PLR, and MLR were not associated with OS or DFS. Similarly, changes in these indices during preoperative chemotherapy were not associated with OS or DFS. Two exploratory subgroup associations were identified: lower preoperative PLR was associated with increased severe postoperative morbidity after major hepatectomy, and greater PLR reduction during preoperative chemotherapy was associated with a higher rate of complete pathological response. These findings should be interpreted cautiously, particularly given the limited discriminatory performance of the ROC‐derived cutoff values.

Inflammation‐based biomarkers have been investigated extensively as potential prognostic indicators in cancer. Among patients with colorectal cancer and metastatic liver disease, previous studies have reported relationships between systemic inflammatory profiles and outcomes following curative‐intent hepatic resection [8, 13, 14, 15, 16, 17, 18, 19, 20, 21, 22].

NLR is the most extensively studied inflammatory index in CRLM. Several studies have associated elevated NLR with worse survival after liver resection [13, 18, 20, 22, 23], and a meta‐analysis of 18 studies including 3184 patients reported associations with both OS and DFS [23]. In contrast, Hsu et al. observed an independent prognostic association for postoperative NLR, but not for preoperative NLR, in patients with solitary CRLM [26]. Other studies have reported divergent findings, including an association between higher preoperative NLR and improved survival [14]. Taken together, these data suggest that the prognostic relevance of NLR may vary according to patient characteristics, treatment exposure, disease presentation, and the timing of biomarker assessment.

Evidence regarding PLR and MLR in CRLM is more limited and similarly inconsistent. Peng et al. reported associations between higher LMR, lower NLR, and improved survival after hepatectomy [8], whereas Neofytou et al. found that PLR had greater prognostic value than NLR; although none of the three indices was significantly associated with OS or DFS in their preoperative analysis [24].

Recent evidence has also examined PLR as a potential prognostic marker in colorectal cancer. In a systematic review and meta‐analysis of 27 studies involving 13 330 patients, Guo et al. reported that higher PLR was associated with poorer overall survival (HR 1.40, 95% CI 1.21–1.62) as well as shorter disease‐free and recurrence‐free survival [27]. In contrast, neither PLR nor MLR was associated with survival in our cohort, reinforcing the variability of these associations among patients with CRLM.

Differences in cutoff definitions may contribute to the variability observed across studies. NLR values vary substantially across populations, and reported thresholds differ according to demographic characteristics, geographic region, and methodological approaches [28, 29, 30, 31]. Importantly, statistical association does not necessarily imply adequate discriminatory performance. In an analysis of five phase III trials, baseline NLR was associated with survival but showed limited discriminatory performance, with AUCs of 0.49–0.57 for OS and 0.51–0.56 for progression‐free survival [32].

In the present study, ROC‐derived cutoff values also showed limited discrimination, with AUCs ranging from 0.489 to 0.531 for mortality and recurrence. Therefore, the thresholds identified in our cohort should not be considered clinically validated cutoffs. Their use for dichotomization may also have reduced the ability to detect clinically meaningful associations. Future studies should preferentially evaluate inflammatory indices as continuous variables and assess their incremental discriminatory value when combined with established clinicopathological and treatment‐related factors.

In the subgroup of patients undergoing major hepatectomy, lower preoperative PLR was associated with more severe postoperative morbidity, defined as Clavien–Dindo Grade III–IV complications (*p* = 0.021). One possible explanation is that a lower PLR may partly reflect lower circulating platelet counts, as thrombocytopenia has been associated with increased morbidity after hepatectomy [33]. However, this finding should be interpreted cautiously because it was observed in a subgroup analysis and was based on a ROC‐derived dichotomous cutoff with limited discriminatory performance. Further studies are required to determine whether PLR provides independent prognostic information beyond established clinical and surgical risk factors.

In patients who received preoperative chemotherapy, longitudinal changes in NLR, PLR, and MLR were not related to OS or DFS. A different pattern was observed for PLR, for which the magnitude of change was associated with complete pathological response (*p* = 0.002). Complete pathological response occurred in 20.8% of patients with ΔPLR ≤ −65.74, compared with 6.8% among those with ΔPLR > −65.74. Because ΔPLR was defined as the preoperative value minus the prechemotherapy value, increasingly negative values indicate greater reductions in PLR. Accordingly, greater decreases in PLR were associated with a higher frequency of complete pathological response. This observation is exploratory and requires validation in independent patient cohorts. The mechanisms underlying this association cannot be determined from the present data.

Findings from recent studies in metastatic colorectal cancer also highlight the potential relevance of evaluating inflammatory biomarkers over time. A 2025 systematic review and meta‐analysis of 19 studies comprising 4422 patients reported associations between higher PLR and shorter OS (HR 1.18, 95% CI 1.03–1.35) and progression‐free survival (HR 1.28, 95% CI 1.03–1.60), although the magnitude of these associations differed among clinical subgroups [34]. Wang et al. reported that preoperative NLR was associated with prognosis in patients undergoing upfront surgery for CRLM, but not in those receiving neoadjuvant treatment, suggesting that systemic therapy may influence the prognostic interpretation of baseline NLR [35]. Similarly, Librenjak et al. found that post‐treatment NLR provided prognostic information that was not fully captured by baseline measurements in patients receiving systemic therapy [36]. Despite these findings, the clinical utility of longitudinal inflammatory biomarkers remains incompletely established, and their interpretation may vary according to the specific biomarker, timing of measurement, treatment setting, and outcome evaluated.

The biological basis of the association between changes in PLR and pathological response remains unclear. Platelets interact with tumor cells, immune cells, and the metastatic microenvironment and may influence tumor progression beyond their role in hemostasis [37, 38]. However, our study did not assess platelet function, immune‐cell activity, cytokine signaling, or other mechanistic pathways.

The lack of consistent associations across inflammatory indices may also reflect the clinical heterogeneity of CRLM. Tumor burden, timing of metastatic presentation, extent of liver resection, response to systemic therapy, chemotherapy regimen, treatment duration, and the interval between chemotherapy and surgery may all influence both prognosis and circulating inflammatory parameters. A single peripheral blood measurement may therefore be insufficient to capture the complex interaction between systemic inflammation, tumor biology, and treatment response.

This study has several limitations. Its retrospective, single‐center design introduces potential selection and confounding biases and may limit generalizability. The cohort was heterogeneous regarding disease characteristics, extent of resection, and exposure to preoperative chemotherapy. In addition, the ROC‐derived cutoff values showed limited discriminatory performance and should not be regarded as clinically validated thresholds. Although longitudinal changes were evaluated, their underlying biological mechanisms were not investigated. Residual confounding cannot be excluded. Furthermore, the number of subgroup and exploratory comparisons increases the possibility of chance findings, particularly for associations identified within restricted patient populations. Additionally, other scores such as the modified Glasgow Prognostic Score could not be calculated due to incomplete preoperative C‐reactive protein and serum albumin data. These limitations reinforce the need for prospective, multicenter validation of the observed associations.

## 5 Conclusion

Preoperative NLR, PLR, and MLR were not associated with overall or disease‐free survival in patients undergoing curative‐intent liver resection for colorectal liver metastases. However, lower preoperative PLR was associated with increased severe postoperative morbidity among patients undergoing major hepatectomy. Furthermore, a greater reduction in PLR following preoperative chemotherapy was associated with a higher rate of complete pathological response. These exploratory findings should be interpreted cautiously and do not support the use of inflammatory indices as standalone biomarkers for clinical decision‐making. Prospective, multicenter studies are warranted to validate these associations and determine whether longitudinal inflammatory indices provide clinically relevant information when integrated with established clinicopathological and treatment‐related factors.

## Author Contributions

**Rider May Cedro:** conceptualization, investigation, writing – original draft, methodology, writing – review and editing, formal analysis, project administration. **Gilton Marques Fonseca:** conceptualization, investigation, methodology, writing – review and editing, formal analysis, project administration, supervision. **Fabíola Trocoli Novaes:** investigation. **José Donizeti Meira Júnior:** investigation. **Vagner Birk Jeismann:** supervision, investigation, writing – review and editing. **Fabio Ferrari Makdissi:** supervision, writing – review and editing. **Jaime Arthur Pirolla Kruger:** supervision, writing – review and editing. **Fabricio Ferreira Coelho:** investigation, formal analysis, writing – review and editing. **Paulo Herman:** conceptualization, methodology, writing – review and editing, project administration, supervision.

## Funding

The authors have nothing to report.

## Conflicts of Interest

The authors declare no conflicts of interest.

## Supporting information


**Table S1:** Distribution of age at surgery in the study cohort.
**Table S2:** Correlation between age and preoperative inflammatory indices.
**Table S3:** Preoperative inflammatory indices according to age group.
**Table S4:** Cox proportional‐hazards models evaluating continuous inflammatory indices adjusted for age and sex.
**Table S5:** Cox proportional‐hazards models evaluating continuous inflammatory indices adjusted for age and sex.
**Table S6:** Interaction analyses evaluating age and sex as potential effect modifiers of the association between inflammatory indices and survival.
**Table S7:** Distribution of patients according to E‐AHPBA metastatic timing.
**Table S8:** Preoperative inflammatory indices according to E‐AHPBA metastatic timing.
**Table S9:** Cox proportional‐hazards models evaluating inflammatory indices according to E‐AHPBA metastatic timing.

## Data Availability

The data that support the findings of this study are available from the corresponding author upon reasonable request. The data are not publicly available due to patient privacy and ethical restrictions.
