# Supplementary material for: Inflammatory Markers and Outcomes After Liver Resection for Colorectal Liver Metastases
Source: Cancer Rep (Hoboken). 2026 Nov 3;9(11):e70725. doi: 10.1002/cnr2.70725 (PMC13729089; doi:10.1002/cnr2.70725)
Supplement: Supplementary file 1 [file CNR2-9-e70725-s001.docx]

**SUPPLEMENTARY MATERIAL**

**Additional analyses of preoperative inflammatory indices according to age, sex, and timing of colorectal liver metastases**

# **1. STATISTICAL METHODS**

These exploratory analyses were performed in response to the reviewer’s request to further evaluate the relationship between preoperative inflammatory indices and patient age, sex, and metastatic timing. The analyses used the same eligible study population described in the main manuscript (n=380). The predefined inclusion and exclusion criteria were retained: adults with colorectal liver metastases undergoing curative-intent liver resection were eligible, whereas patients with extrahepatic metastatic disease, previous two-stage hepatectomy, previous portal vein embolization, or unavailable or insufficient medical records were excluded.

Preoperative neutrophil-to-lymphocyte ratio (NLR), platelet-to-lymphocyte ratio (PLR), and monocyte-to-lymphocyte ratio (MLR) were calculated from the absolute blood-cell counts obtained before liver resection, according to the definitions used in the main manuscript. One patient had invalid preoperative neutrophil, lymphocyte, and monocyte values coded as −99; this record was retained in the overall cohort but excluded from analyses requiring calculation of NLR, PLR, or MLR.

Continuous variables were summarized as medians with interquartile ranges (IQRs) when appropriate. Associations between age and inflammatory indices were assessed using Spearman rank correlation. Pearson correlation coefficients are also reported for completeness. Comparisons between two groups were performed using the Mann–Whitney U test, and comparisons among the three E-AHPBA metastatic-timing categories were performed using the Kruskal–Wallis test. All tests were two-sided, with p<0.05 considered statistically significant.

For exploratory prognostic analyses, Cox proportional-hazards models evaluated each inflammatory index as a continuous variable. Additional models adjusted for age and sex. To assess effect modification, interaction terms between each inflammatory index and age or sex were evaluated. For metastatic timing, models included E-AHPBA category, the inflammatory index, and index-by-timing interaction terms. Synchronous disease was used as the reference category. Overall survival (OS) and disease-free survival (DFS) were defined as in the main manuscript.

# **2. AGE AND PREOPERATIVE INFLAMMATORY INDICES**

Age at surgery was 60.9 ± 10.2 years, with a median of 62 years (IQR, 55–68; range, 26–84). When age was evaluated as a continuous variable, no significant correlation was observed with NLR or MLR. PLR showed a weak inverse correlation with age.

**Table S1.** Distribution of age at surgery in the study cohort.

| **Variable** | **n** | **Mean ± SD** | **Median (IQR)** | **Range** |
| --- | --- | --- | --- | --- |
| Age at surgery, years | 380 | 60.9 ± 10.2 | 62 (55–68) | 26–84 |

SD, standard deviation; IQR, interquartile range

**Table S2.** Correlation between age and preoperative inflammatory indices.

| **Index** | **n** | **Pearson r** | **p** | **Spearman ρ** | **p** |
| --- | --- | --- | --- | --- | --- |
| NLR | 379 | −0.047 | 0.362 | −0.021 | 0.679 |
| MLR | 379 | −0.032 | 0.530 | −0.023 | 0.658 |
| PLR | 379 | −0.135 | **0.008** | −0.120 | **0.020** |

NLR, neutrophil-to-lymphocyte ratio; PLR, platelet-to-lymphocyte ratio; MLR, monocyte-to-lymphocyte ratio.

For exploratory stratification, the cohort median age (62 years) was used as the cutoff. Among patients with calculable inflammatory indices, 189 were younger than 62 years and 190 were aged 62 years or older. NLR and MLR did not differ between age groups, whereas PLR was lower among patients aged 62 years or older, with a borderline p-value.

**Table S3.** Preoperative inflammatory indices according to age group.

| **Index** | **Age <62 years, n=189** | **Age ≥62 years, n=190** | **p** |
| --- | --- | --- | --- |
| NLR | 2.00 (1.27–3.01) | 1.96 (1.34–3.07) | 0.917 |
| MLR | 0.35 (0.26–0.50) | 0.35 (0.26–0.50) | 0.962 |
| PLR | 125.71 (87.50–173.27) | 112.75 (76.79–155.69) | **0.050** |

NLR, neutrophil-to-lymphocyte ratio; PLR, platelet-to-lymphocyte ratio; MLR, monocyte-to-lymphocyte ratio. Values are median (IQR). Mann–Whitney U test.

# **3. PROGNOSTIC ANALYSES ADJUSTED FOR AGE AND SEX**

To address whether the prognostic associations of the inflammatory indices were materially affected by age and sex, additional Cox models were fitted with each index as a continuous variable and adjusted for age and sex. These analyses were exploratory and were not part of the prespecified primary prognostic analysis.

In these exploratory adjusted models, NLR and PLR were not significantly associated with OS or DFS. MLR showed a statistically significant association with both OS and DFS after adjustment for age and sex. Because the primary analyses in the manuscript used ROC-derived categorical thresholds and because the ROC analyses demonstrated limited discriminatory performance, these adjusted continuous-variable findings should be considered exploratory rather than as evidence supporting a clinically validated prognostic threshold.

**Table S4.** Cox proportional-hazards models evaluating continuous inflammatory indices adjusted for age and sex.

| **Outcome** | **Index** | **HR** | **95% CI** | **p** |
| --- | --- | --- | --- | --- |
| OS | NLR | 1.059 | 0.986–1.137 | 0.114 |
|  | MLR | 1.521 | 1.077–2.150 | **0.017** |
|  | PLR | 1.001 | 1.000–1.002 | 0.090 |
| DFS | NLR | 1.038 | 0.973–1.108 | 0.255 |
|  | MLR | 1.624 | 1.149–2.297 | **0.006** |
|  | PLR | 1.001 | 1.000–1.002 | 0.116 |

HR, hazard ratio; CI, confidence interval; OS, overall survival; DFS, disease-free survival.

# **4. SEX AND PREOPERATIVE INFLAMMATORY INDICES**

Among patients with calculable inflammatory indices, NLR and MLR were higher in male than in female patients, whereas PLR did not differ significantly between sexes.

**Table S5.** Cox proportional-hazards models evaluating continuous inflammatory indices adjusted for age and sex.

| **Index** | **Male**  **(n=210)** | **Female**  **(n=169)** | **p** |
| --- | --- | --- | --- |
| NLR | 2.13 (1.35–3.25) | 1.70 (1.18–2.67) | **0.012** |
| MLR | 0.40 (0.29–0.55) | 0.30 (0.24–0.41) | **<0.001** |
| PLR | 115.63 (76.13–163.38) | 122.50 (85.22–165.33) | 0.248 |

Values are median (IQR). Mann–Whitney U test. The overall cohort contains 210 male and 170 female patients; one female patient had invalid preoperative differential-count values and was therefore excluded from index-specific analyses.

# **5. INTERACTION ANALYSES ACCORDING TO AGE AND SEX**

Formal interaction analyses were performed to evaluate whether age or sex modified the association between each inflammatory index and survival. Age was modeled continuously, and sex was modeled as male versus female. The models also included the main effects of the inflammatory index, age, and sex.

Age significantly modified the association between NLR, MLR, and PLR and OS, and the associations between NLR and MLR and DFS. No significant interaction between sex and any inflammatory index was identified for either OS or DFS. Because interaction terms involving continuous biomarkers and age are scale-dependent and the subgroup analyses are exploratory, these findings should be interpreted cautiously and should not be used to establish age-specific clinical cutoffs.

**Table S6.** Interaction analyses evaluating age and sex as potential effect modifiers of the association between inflammatory indices and survival.

| **Outcome** | **Index** | **p for index × age** | **p for index × sex** |
| --- | --- | --- | --- |
| OS | NLR | **0.008** | 0.564 |
|  | MLR | **0.035** | 0.602 |
|  | PLR | **0.003** | 0.761 |
| DFS | NLR | **0.032** | 0.412 |
|  | MLR | **0.027** | 0.944 |
|  | PLR | 0.160 | 0.184 |

P-values are from the corresponding interaction terms in Cox proportional-hazards models.

# **6. METASTATIC TIMING ACCORDING TO E-AHPBA CLASSIFICATION**

According to the E-AHPBA consensus criteria, CRLM were categorized as synchronous when identified on or before the diagnosis of the primary colorectal tumor, early metachronous when diagnosed within 12 months after the primary tumor, and late metachronous when diagnosed more than 12 months after the primary tumor. The resulting distribution was 56 patients with synchronous disease (14.7%), 214 with early metachronous disease (56.3%), and 110 with late metachronous disease (28.9%). No statistically significant differences in NLR, MLR, or PLR were observed across the three E-AHPBA metastatic-timing categories.

**Table S7.** Distribution of patients according to E-AHPBA metastatic timing.

| **E-AHPBA metastatic timing** | **n** | **%** |
| --- | --- | --- |
| Synchronous | 56 | 14.7 |
| Early metachronous | 214 | 56.3 |
| Late metachronous | 110 | 28.9 |
| Total | 380 | 100.0 |

**Table S8.** Preoperative inflammatory indices according to E-AHPBA metastatic timing.

| **Index** | **Synchronous**  **(n=56)** | **Early metachronous**  **(n=213)** | **Late metachronous**  **(n=110)** | **p** |
| --- | --- | --- | --- | --- |
| NLR | 2.13 (1.44–3.08) | 1.80 (1.24–2.86) | 2.19 (1.42–3.21) | 0.086 |
| MLR | 0.36 (0.28–0.47) | 0.35 (0.26–0.50) | 0.36 (0.26–0.50) | 0.789 |
| PLR | 118.60 (82.96–169.18) | 115.87 (82.00–165.63) | 118.59 (78.24–158.74) | 0.953 |

Values are median (IQR). Kruskal–Wallis test. One patient in the early metachronous group had invalid preoperative differential-count values.

# **7. PROGNOSTIC ANALYSES ACCORDING TO METASTATIC TIMING**

To determine whether metastatic timing modified the prognostic association of the inflammatory indices, Cox models were fitted including the E-AHPBA timing category, the continuous inflammatory index, and index-by-timing interaction terms. Synchronous disease was used as the reference category.

No statistically significant interaction was identified between metastatic timing and NLR, MLR, or PLR for either OS or DFS. Thus, although individual timing strata showed occasional nominal associations, the formal interaction analyses did not provide statistical evidence that the prognostic association of the inflammatory indices differed according to E-AHPBA metastatic timing.

**Table S9.** Cox proportional-hazards models evaluating inflammatory indices according to E-AHPBA metastatic timing.

| **Outcome** | **Index** | **HR in synchronous group** | **95% CI** | **p** | **p for interaction** |
| --- | --- | --- | --- | --- | --- |
| OS | NLR | 1.168 | 0.946–1.442 | 0.154 | 0.147 |
|  | MLR | 4.249 | 0.892–20.237 | 0.075 | 0.141 |
|  | PLR | 1.003 | 0.998–1.007 | 0.239 | 0.769 |
| DFS | NLR | 1.023 | 0.974–1.074 | 0.819 | 0.674 |
|  | MLR | 3.455 | 0.807–14.787 | 0.095 | 0.389 |
|  | PLR | 1.001 | 0.999–1.003 | 0.598 | 0.969 |

HR, hazard ratio; CI, confidence interval. The p-value for interaction is the joint test of the index-by-early-metachronous and index-by-late-metachronous interaction terms.

# **SUPPLEMENTARY RESULTS SUMMARY**

Additional exploratory analyses were performed to address potential modification of the prognostic performance of preoperative inflammatory indices by age, sex, and metastatic timing. Age was not significantly correlated with NLR or MLR, whereas PLR showed a weak inverse correlation with age. NLR and MLR were higher in male patients, while PLR did not differ significantly according to sex. In Cox models adjusted for age and sex, NLR and PLR were not significantly associated with OS or DFS, whereas MLR showed significant associations with both outcomes. Interaction analyses suggested that age may modify the associations of NLR, MLR, and PLR with OS and of NLR and MLR with DFS, whereas no significant effect modification by sex was observed. According to the E-AHPBA classification, inflammatory indices did not differ significantly across synchronous, early metachronous, and late metachronous disease. Furthermore, no significant interaction between metastatic timing and NLR, MLR, or PLR was observed for OS or DFS.
